# Identification of losses to follow-up in a community-based antiretroviral therapy clinic in South Africa using a computerized pharmacy tracking system

**DOI:** 10.1186/1471-2334-10-329

**Published:** 2010-11-15

**Authors:** Mweete D Nglazi, Richard Kaplan, Robin Wood, Linda-Gail Bekker, Stephen D Lawn

**Affiliations:** 1 The Desmond Tutu HIV Centre, Institute of Infectious Disease and Molecular Medicine, Department of Medicine, Faculty of Health Sciences, University of Cape Town, South Africa; 2 The International Union Against Tuberculosis and Lung Disease; 3 Department of Infectious and Tropical Diseases, London School of Hygiene and Tropical Medicine, London, United Kingdom

## Abstract

**Background:**

High rates of loss to follow-up (LTFU) are undermining rapidly expanding antiretroviral treatment (ART) services in sub-Saharan Africa. The intelligent dispensing of ART (iDART) is an open-source electronic pharmacy system that provides an efficient means of generating lists of patients who have failed to pick-up medication. We determined the duration of pharmacy delay that optimally identified true LTFU.

**Methods:**

We conducted a retrospective cross-sectional study of a community-based ART cohort in Cape Town, South Africa. We used iDART to identify groups of patients known to be still enrolled in the cohort on the 1^st ^of April 2008 that had failed to pick-up medication for periods of ≥ 6, ≥ 12, ≥ 18 and ≥ 24 weeks. We defined true LTFU as confirmed failure to pick up medication for 3 months since last attendance. We then assessed short-term and long-term outcomes using a prospectively maintained database and patient records.

**Results:**

On the date of the survey, 2548 patients were registered as receiving ART but of these 85 patients (3.3%) were found to be true LTFU. The numbers of individuals (proportion of the cohort) identified by iDART as having failed to collect medication for periods of ≥6, ≥12, ≥18 and ≥24 weeks were 560 (22%), 194 (8%), 117 (5%) and 80 (3%), respectively. The sensitivities of these pharmacy delays for detecting true LTFU were 100%, 100%, 62.4% and 47.1%, respectively. The corresponding specificities were 80.7%, 95.6%, 97.4% and 98.4%. Thus, the optimal delay was ≥12 weeks since last attendance at this clinic (equivalent to 8 weeks since medication ran out). Pharmacy delays were also found to be significantly associated with LTFU and death one year later.

**Conclusions:**

The iDART electronic pharmacy system can be used to detect patients potentially LTFU and who require recall. Using a short a cut-off period was too non-specific for LTFU and would require the tracing of very large numbers of patients. Conversely prolonged delays were too insensitive. Of the periods assessed, a ≥12 weeks delay appeared optimal. This system requires prospective evaluation to further refine its utility.

## Background

Antiretroviral therapy (ART) has become much more widely available in resource-limited countries with a high burden of HIV/AIDS. Four million people were estimated to be receiving ART in low- or middle- income countries by the end of 2008, of whom 2.9 million were in sub-Saharan Africa and 701,000 were in South Africa alone [1]. Success in scale-up, however, is being tempered by the fact that escalating case-loads of patients attending individual clinics present a major challenge to the effective retention of patients in care.

Low levels of retention particularly threaten to undermine ART programmes in sub-Saharan Africa. Here, 8%-26% of patients die in the first year of ART [2] and a further proportion may be lost to follow-up (LTFU), with combined attrition rates of approximately 40% at 2 years in many programmes [3]. Identifying and tracing patients who are potentially LTFU is essential to maintain programme quality. However, the human resources needed for this are very limited and development of information systems that permit the most effective deployment of these resources would be of great benefit.

Pharmacy-based records of collection of medication by patients can be used as a system for early identification of patients who are potentially LTFU [4]. iDART, for example, is a computerized software system that requires no licence and is freely available to download at URL http://www.cell-life.org/idart/download. This system allows clinic management teams to generate lists of patients who failed to pick up medication and who can then be traced in the community. However, it is not known what delay of pharmacy pick-ups optimally identifies true programme losses to follow-up.

We therefore conducted a study at a community-based ART clinic in Cape Town, South Africa, using pharmacy dispensing data recorded by the iDART system to examine the relationship between the duration of the delay in scheduled pharmacy pick-ups of medication and LTFU. We hypothesized *a priori *that too short a delay would be too non-specific and would inadvertently lead to the tracing of large numbers of patients who were not LTFU. Conversely, we hypothesized that too prolonged a delay would fail to identify a proportion of patients LTFU who did require tracing.

## Methods

### Setting

This is a well characterized ART service [5-7] in a poor peri-urban area in Cape Town, South Africa. The Desmond Tutu HIV Centre began providing ART at this service in September 2002 and by April 2008, 3384 patients had started ART. The clinic is supported by an on-site pharmacy staffed by pharmacists and pharmacy assistants. Patients routinely pick up drug supplies for one month, although for various reasons a minority may be given a 2-months supply. Electronic pharmacy pick-up data by patients has been recorded since the introduction of iDART in February 2007.

### Data sources

Data for this analysis were obtained from the following 3 sources: (1) a prospectively maintained ART cohort database that is updated weekly and contains outcomes derived from patient notes and data forms supplied by the ART service; (2) iDART electronic pharmacy records of ART regimens and dispensing dates; (3) individual patient records.

### Definitions

In keeping with the majority of other literature [3], true losses to follow up (LTFU) were patients who had failed to attend the clinic for ≥12 weeks and who were not known to have died or been transferred to another ART clinic. Death referred to all-cause mortality notified by peer counselors after home visits, relatives/family members and hospitals. Previous data suggest that differentiation between deaths and LTFU using these sources is possible [6,7]. Transfers-out referred to patients receiving ART whose care was transferred to another clinic.

### Study design

This was a retrospective cross-sectional survey of patients enrolled on treatment on the 1^st ^of April 2008, with ascertainment of the reasons for failure to collect medication both at the time of the survey as well as one year after the survey. The ART cohort database was first used to define the patients who were still enrolled and should be receiving ART in the cohort. Within this active treatment cohort, patients were identified as potential LTFU using the iDART dispensing system based on time since last attendance at the clinic to collect medication. Patients failing to collect medication were categorized into four groups based on the duration of the delay: i) ≥6 weeks ii) ≥12 weeks and iii) ≥18 weeks and iv) ≥24 weeks.

Patient records and the cohort database were then used to determine the reasons for failure to pick up medication by these 4 patient groups. The outcomes of the treatment cohort who were included in the cross-sectional survey were assessed at that time (short-term outcomes). In addition, the outcomes of these same individuals were determined at a time-point one year later (long-term outcomes). The relationship between the period of delay in pharmacy pick-ups and the outcomes of the patients was then determined.

Ethical approval for this study was obtained from the Research Ethics Committees of the University of Cape Town and the International Union Against Tuberculosis and Lung disease.

### Statistical analyses

The study cohort was defined as patients known to be receiving ART at the 1^st ^of April 2008. Descriptive statistics were used to characterize the cohort. Proportions were used for categorical variables. Means, medians, standard deviations and interquartile ranges were used for continuous variables as appropriate. The proportion of each of the true outcomes of patients at each of the delayed periods and associated confidence intervals were determined. For each period of delay, we calculated the sensitivity, specificity, positive predictive value and negative predictive value for predicting LTFU and associated confidence intervals. Confidence intervals were calculated using exact binomial techniques. Statistical analysis was performed using STATA version 10.0 (College Station, Texas, USA).

## Results

### Treatment cohort

Between September 2002 when the clinic was first opened and the time of the cross-sectional survey in April 2008, a total of 3384 patients had initiated ART. During this period, 334 (9.9%) were LTFU, 249 (7.4%) were transferred out, and 253 (7.5%) had died. This left 2548 patients who were registered as still receiving ART and therefore formed the patient cohort at the time of this cross-sectional survey.

The characteristics of the patients in the study cohort (n = 2548) at the time of ART initiation were as follows: a majority (86%) of patients were ART-naïve, most were female (67%) and the median age was 32 years (interquartile range [IQR], 27-38). Immunodeficiency was advanced with a median blood CD4 lymphocyte count of 124 cells/μL (IQR, 63-192). Disease was categorized as World Health Organization (WHO) clinical stage III in 52.4% and stage IV in 21.5%. These patients had been receiving ART within this service for a median of 1.9 years (IQR, 1.0-2.9).

### Patients identified as potentially LTFU

**Figure 1:**
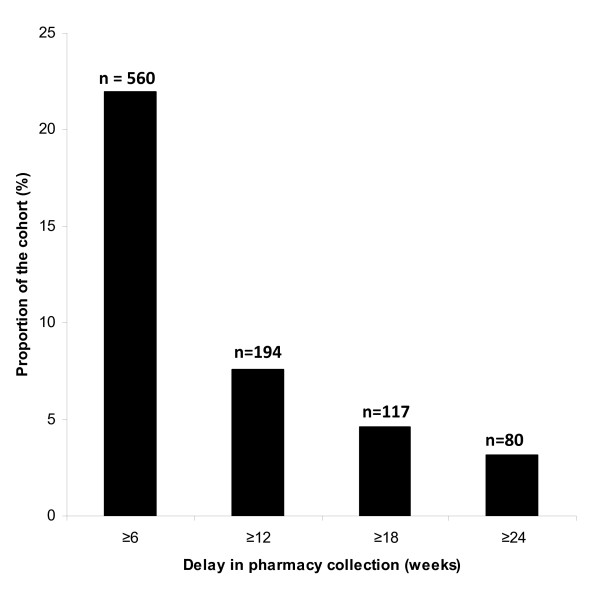
**Proportions of the antiretroviral treatment (ART) cohort identified as having had missed pharmacy visits for periods of ≥ 6, ≥ 12, ≥ 18 or ≥ 24 weeks using the iDART pharmacy tracking system**. Absolute numbers of patients in each category are shown above each bar.

The iDART pharmacy tracking system was used to identify groups of patients within cohort that failed to collect ART for periods of ≥6, ≥12, ≥18 and ≥24 weeks. Those missing pharmacy visits for ≥6 weeks represented 22% of the whole cohort and the number of patients was 2.9 times higher than that identified using the ≥12 weeks cut-off. More prolonged pharmacy delay cut-offs identified substantially fewer patients as potential LTFU (Figure 1).

### True outcomes of patients identified by the iDART system

We next explored the underlying reasons for pharmacy delays other than LTFU (Table 1). Of the large number of patients identified as having a ≥ 6 weeks pharmacy delay, just 15.2% were true LTFU and a large majority of the remaining patients were actively receiving ART (Table 1). Of patients identified using the ≥ 12, ≥ 18 and ≥ 24 weeks cut-offs, the proportions of patients with each outcome were very similar. The proportions who were true LTFU ranged from 44-50%. Most other patients were actually still in care and either receiving treatment (range, 23-25%) or had temporarily interrupted treatment (18-22%). A smaller proportion of these patients were found to have died (4%-6%).

**Table 1:** Outcomes of patients identified iDART pharmacy tracking system using four different delays in pharmacy pick-ups.

Weeks delay of pharmacy pick-ups								
	≥6		≥12		≥18		≥24	
	
	N	Proportion (95% CI)	N	Proportion (95% CI)	N	Proportion (95% CI)	N	Proportion (95% CI)
True LTFU	85	15.2 (12.2-18.2)	85	43.8 (36.8-50.9)	53	45.3 (36.1-54.5)	40	50.0 (38.8-61.2)
Deaths	10	1.8 (0.7-2.9)	8	4.1 (1.3-6.9)	7	6.0 (1.6-10.3)	3	3.8 (-0.5-8.0)
Transfers-out	19	3.4 (1.9-4.9)	14	7.2 (3.5-10.9)	5	4.3 (0.6-8.0)	5	6.3 (0.8-11.7)
On treatment	404	72.1 (68.4-75.9)	45	23.2 (17.2-29.2)	29	24.8 (16.8-32.7)	18	22.5 (13.1-31.9)
In clinic off treatment	42	7.5 (5.3-9.7)	42	21.6 (15.8-27.5)	23	19.7 (12.3-27.0)	14	17.5 (9.0-26.0)
								

**Total**	**560**	**22.0 (20.4-23.6)**	**194**	**7.6 (6.6-8.6)**	**117**	**4.6 (3.8-5.4)**	**80**	**3.1 (2.5-3.8)**

LTFU, loss to follow-up; N, number; CI, confidence intervals

### Sensitivity and specificity of pharmacy delays

In total, there were 85 patients who were true LTFU, representing 3.3% of the overall cohort. We calculated the sensitivities, specificities and positive and negative predictive values of the ≥6, ≥12, ≥18 and ≥24 week iDART pharmacy delay periods (Table 2). Delays of the ≥6 and ≥12 weeks were 100% sensitivity for true LTFU whereas more prolonged delays showed much lower sensitivities. However, the specificity of the ≥12 week delay was substantially greater than that of the ≥6 week delay (Table 2).

**Table 2:** Raw data, sensitivity, specificity and positive predictive value and negative predictive value for the four different delays in pharmacy pick-ups

Delays of pharmacy pick-ups, weeks*	a	b	c	d	Sensitivity %(95% CI)	Specificity %(95% CI)	PPV % (95% CI)	NPV % (95% CI)
**≥6**	85	475	0	1988	100.0 (95.8-100.0)	80.7 (79.1-82.3)	15.2 (12.2-18.2)	100.0 (99.8 -100.0)
**≥12**	85	109	0	2354	100.0 (95.8-100.0)	95.6 (94.7-96.4)	43.8 (36.8-50.9)	100.0 (99.8-100.0)
**≥18**	53	64	32	2399	62.4 (51.2-72.6)	97.4 (96.7-98.0)	45.3 (36.1-54.5)	98.7 (98.1-99.1)
**≥24**	40	40	45	2423	47.1 (36.1-58.2)	98.4 (97.8-98.8)	50.0 (38.8-61.2)	98.2 (97.6-98.7)

*Pharmacy delays detected by iDART were compared to true LTFU status; a, true positives; b, false positives; c, false negatives; d, true negatives; PPV, positive predictive value; NPV, negative predictive value; %, percentage and CI, confidence intervals

### Outcomes at one year

**Figure 2:**
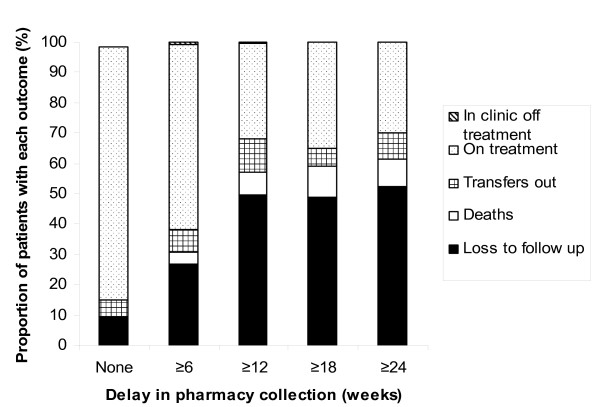
**Long-term outcomes (one year after the cross-sectional survey) of patients who either had no pharmacy delay (n = 1988) or who delayed collecting antiretroviral therapy (ART) for ≥6, ≥12, ≥18 or ≥24 weeks**. For each group, the proportions of patients with each outcome are shown.

We next determined the status of the same 4 groups of patients at a time-point one year after the cross-sectional survey (Figure 2) and compared these with the outcomes of patients who had no pharmacy delay at the time of the survey (n = 1988). Twenty two patients who were originally LTFU had returned to care within the year of follow-up. However, much higher proportions of those with delays of ≥6, ≥12, ≥18 and ≥24 weeks were designated as LTFU one year later compared to patients with no pharmacy delay [27.0% (151/560), 49.5% (96/194), 48.7% (57/117) and 52.5% (42/80) versus 9.5% (189/1988), respectively P < 0.001 for all comparisons] (Figure 2). Similarly, higher proportions of those with pharmacy delays had died after one year [3.8% (21/560), 7.7% (15/194), 10.3% (12/117) and 8.8% (7/80) versus 0.02% (34/1988), respectively; P < 0.02 for comparisons of ≥12 weeks group and ≥18 weeks groups with no delay group]. Thus, detection of pharmacy delays was prognostic of poor long-term outcomes with regard to LTFU and mortality.

## Discussion

Practical solutions are urgently needed to address the challenge of low rates of patient retention in many ART programmes in sub-Saharan Africa [8]. A critical issue is the need to rapidly identify patients who have missed appointments so that patient tracing interventions can be deployed to re-engage patients as soon as possible in care. Using the iDART computerized pharmacy tracking system, we determined the optimal period of delay in pharmacy pick-ups that best identified true LTFU. A ≥6 weeks cut-off would result in the tracing of very large numbers of patients of whom only 15% (approximately 1 in 7) would be true LTFU. Use of cut-offs of ≥12, ≥18 and ≥24 weeks, however, would greatly reduce the numbers of patients who would require tracing, but the ≥12 weeks cut-off was found to be optimal with a sensitivity of 100.0% and a specificity of 95.6%.

Patients who are LTFU should ideally be re-engaged back in care as soon as possible and thus we included within this analysis an examination of a short delay in pharmacy pick-ups (≥6 weeks, typically representing 2 weeks since medication supply ran out) which had high sensitivity. However, one fifth of the cohort had failed to collect medication for ≥6 weeks and yet only a small minority was actually LTFU. Shorter delays than this period would be even more non-specific and the associated work-load associated with tracing all these patients would not be feasible in this service.

In keeping with most other literature from sub-Saharan Africa, we defined true LTFU as patients who had failed to attend for a period of ≥3 months [3]. However, we hypothesized that use of pharmacy delays longer than this period might identify smaller groups of patients with a high yield of LTFU, thereby minimizing the numbers of patients needing to be traced. Indeed, use of increasingly prolonged cut-offs of ≥12, ≥18 and ≥24 weeks was associated with sequential reductions in the numbers of patients that would require tracing. However, more prolonged delays of ≥18 and ≥24 weeks had substantially reduced sensitivity for LTFU despite higher specificity. Thus, on the basis of trade-off between sensitivity and specificity, we identified the ≥12 weeks delay as the optimal cut-off among the four periods assessed. This is entirely consistent with the optimal delay for identifying LTFU found in a study conducted in Zambia (56 days since medication ran out) [9].

We also ascertained outcomes of each of the same 4 groups of patients one year after the time of the cross-sectional study and compared them with the outcomes of patients for whom no pharmacy delay was detected. Approximately one quarter of patients with pharmacy delays of ≥6 weeks and one half of the patients with pharmacy delays of ≥12, ≥18 and ≥24 weeks were LTFU at this time-point compared to just 9.9% of those with no pharmacy delay. Moreover, higher proportions of those with pharmacy delays had died by one year follow-up. This indicates that the detection of pharmacy delays using the iDART system can be used to identify groups of patients who have poor long-term retention and increased mortality risk.

Computerised systems are used to track patients efficiently and are a potential solution for the rapid identification of patients potentially LTFU. However, the utility of these systems depends on user friendliness, affordability, sustainability, stability, security and data ownership. Sites across Africa employ a wide range of electronic information systems to identify patients to be potentially traced [10,11]. The iDART system is non-commercial, user friendly, requires no license and is freely available to download at URL http://www.cell-life.org/idart/download. However, it requires an uninterrupted electricity supply and requires a computer, barcode printer, barcode reader and offsite back-up such a flash memory stick, cell phone, email or internet connection. This system has advantages over other known electronic information systems used in Haiti, Kenya, Malawi and Zambia in terms of its low cost, user friendliness, minimal staff training requirements and sustainability [11].

The strengths of this study include the fact that iDART is a relatively simple retention measure that could potentially be implemented in settings that are able to support the required infrastructure. In 2009, the system was being successfully used in over 35 sites, mainly in South Africa but also in other countries. The cohort studied is within the South African public sector system and is very well characterized with good quality data on patient outcomes. Limitations include the retrospective design of the study and that the impact of any existing interventions active within this clinic on one year outcomes is unknown. Although this initial cross-sectional study explores the association between LTFU and one key variable (i.e. delays in pharmacy pick-ups), multiple factors may be associated with LTFU and these factors may vary with duration of ART. The important findings of this initial cross-sectional study have been used to devise a long-term prospective study in which the complexities of predicting LTFU can be further refined. This may enable more sophisticated algorithms to identify patients who are potentially LTFU to be developed in due course. Outcomes may differ in clinics with different ART dispensing patterns. Although this study suggests that ≥12 weeks since last clinic attendance to pick-up medication is the optimal definition of LTFU, the choice of optimal delay in other settings may depend on the resources available.

## Conclusions

In summary, the iDART electronic pharmacy system can best be used to identify patients potentially LTFU and with high risk of long-term LTFU or death by detecting those who have failed to collect medication for a period of ≥12 weeks. Approximately one half of such patients were true LTFU and use of this system may be used to trigger patient tracing. Use of this system should be evaluated prospectively in tandem with patient tracing interventions to assess the effects on both short-term and long-term outcomes.

## Competing interests

The authors declare that they have no competing interests.

## Authors' contributions

RK, RW, L-GB and SDL designed the study. MDN collected the data and did the analyses with input from RK and SDL. RK, RW and L-GB developed the research infra-structure and implemented the iDART system. MDN and SDL wrote the paper with input from all the authors who each approved the final version.

## Pre-publication history

The pre-publication history for this paper can be accessed here:

http://www.biomedcentral.com/1471-2334/10/329/prepub
